# Shock and Collapse Resulting from Dental Operations

**Published:** 1892-06

**Authors:** John S. Marshall

**Affiliations:** 9 Jackson Street; Chicago, Ill.


### International Dental Journal.

Vol. XIII. June, 1892. No. 6.

### Original Communications.[1]

### SHOCK AND COLLAPSE RESULTING FROM DENTAL OPERATIONS.[2]

BY JOHN S. MARSHALL, M.D., CHICAGO, ILL.

Mr. President and Gentlemen of the Odontological Society of New York,—When invited by the chairman of your Executive Committee, Dr. J. Morgan Howe, to prepare a paper for your society, I was for several days thereafter in the "slough of despond," from the fact that I despaired of finding a theme which had not already been worn threadbare by the numerous writers upon dental subjects. I was, moreover, ambitious to introduce a new subject; but failing in this, to present a few new thoughts upon some old topic, or, at least, a new application of some old principle.

I am indebted, at this time, for the suggestion of my subject to my colleague, Dr. Allport. The question is one which has not been so constantly before the profession as some others, and for this reason, I trust, will prove a more acceptable and interesting topic for discussion.

It is my purpose therefore to lay before you this evening a few thoughts upon "Shock and Collapse resulting from Dental Operations."

---

[1] The editor and publishers are not responsible for the views of authors of papers published in this department, nor for any claim to novelty, or otherwise, that may be made by them. No papers will be received for this department that have appeared in any other journal published in the country.

[2] Read at a meeting of the New York Odontological Society, March 15, 1892.

If, when I have finished, my critics find it necessary to amputate some unsightly excrescence, to probe to the bottom some deep-seated and foul-smelling ulceration, or possibly to extirpate the heart of the subject, I bespeak only that the scalpel be keen, and the hand that wields it steady. Anæsthetics may be omitted.

### SHOCK.

*Definition.*—Shock is a good old Saxon word, meaning, to shake,—to shake with violence. As applied to medicine and surgery, it comprehends "sudden or instantaneous depression of organic, nervous, or vital power, often with more or less perturbation of body and mind, passing either into reaction or into fatal sinking; occasioned by the nature, severity, or extent of an injury, or by overwhelming moral calamity." (Copland, in Dunglison's "Medical Dictionary.") Another definition is as follows: "A condition of sudden depression of the whole functions of the body, due to powerful impressions upon the system by physical injury or mental emotion. Its more obvious manifestations are signs of lowered activity of the cardiac, respiratory, and sensorial functions; and reduction of surface temperature." (Sir William MacCormac, in Quain's "Dictionary of Medicine.")

### COLLAPSE.

*Definition.*—Collapse comes from the Latin *Collapsus*, meaning, to fall together,—to fall in ruins. Medically, a general prostration or failure of the vital powers. "A complete prostration of strength, either at the commencement or in the progress of the disease." (Dunglison's "Medical Dictionary.") Another authority says, "Collapse is a state of nervous prostration. When it is extreme, the vital functions are in a condition of partial and, sometimes, nearly complete abeyance. It may terminate in death, or be followed by gradual reaction and complete recovery. . . . Collapse and shock have usually been classed together, but it is not accurate to do so. It is true that the ganglionic centres of the medulla oblongata are more or less profoundly involved in both, and that both possess many symptoms in common, dependent upon the derangement of function of one or more of these centres. Some confusion is attributable to the fact that shock is a term applied not only to a state or morbid condition, but to the cause which most frequently produces that condition,—a violent impression or 'shock' to the nervous centres. Collapse arises from many different causes, shock being one, of which collapse may be regarded as a final and extreme degree, and into which it often imperceptibly passes.

"Collapse, on the other hand, may occur under conditions where there has been no antecedent state of shock. Collapse presupposes nervous exhaustion, while shock may instantly appear in a healthy individual." (Sir William MacCormac, in Quain's "Dictionary of Medicine.")

Shock, therefore, may appear instantaneously in healthy individuals, and is caused by fright, extreme mental emotion, accidental injury, or surgical operations; the depression of vital force being slight or severe according to the impression made upon the nerve-centres; the tendency being generally towards reactions, which may take place in a few minutes or not for several hours; while in extreme cases it may terminate fatally.

Collapse, on the other hand, generally manifests itself in persons who have undergone previous prolonged nervous strain which has caused exhaustion; the system is therefore at a disadvantage if called upon to sustain the debilitating effects of disease, or any of the accidental, emotional, or surgical conditions just mentioned. The depression of vital force is more prolonged and extreme, depending upon the strength of the individual and the character and severity of the exciting cause. Reaction sets in less quickly, and complete recovery may require weeks or months to be accomplished.

Collapse, in its most extreme form, following cholera, yellow fever, or shock from severe accident or surgical injury generally, proves fatal. Shock, with severe hemorrhage, often causes fatal collapse.

Prolonged and painful dental operations generally produce considerable depression, and in individuals already in a more or less exhausted condition from disease, overwork of mind or body, emotional causes, and occasionally in chlorotic girls, pregnant women, or those suffering from female complaints, and in frail and delicate children, they may produce alarming symptoms of collapse.

There is in the normal condition of the system a very great difference in the nervous excitability and physical endurance of individuals; while in the same persons under abnormal conditions the individual peculiarities are accentuated. All are less able to endure the shock of accidental or surgical injury, or the extremes of joy or grief, if the physical or mental conditions are below the normal.

It is, therefore, as much the duty of the dentist to look into the general conditions of the health of those who present themselves for operations as it is for the surgeon to do so, before commencing any surgical procedure. Only in extreme cases, when immediate operation seems necessary, as the only chance of saving life, will the intelligent surgeon permit himself to operate upon individuals whose immediate physical condition,—as in shock and collapse,—or whose general health, is greatly impaired, without first making the attempt to recuperate the depressed vitality, and place the patient in the best possible condition for the operation. Failure to appreciate the necessity of such treatment tends to disappointment, or disaster of greater or less moment, either in the success of the operation, the after-effects upon the system, or the life of the patient.

There are but few dental diseases which *per se* are dangerous to life in otherwise healthy individuals, and few operations which can be considered as in any sense dangerous to the same class of persons; and yet the presence of an alveolar abscess or the extraction of a tooth occasionally proves fatal, either from septic infection, a peculiarity in diathesis, or some pre-existent morbid tendency which predisposes to a fatal termination.

On the other hand, painful or prolonged dental operations, like the preparation of a series of exceedingly sensitive cavities of decay, or restoring the contour of the teeth with gold, are very depressing and irritating to the nervous system.

Dr. Litch, in the "American System of Dentistry," vol. iii. p. 71, says, "Operations upon the teeth and associated tissues are often excruciatingly—and in certain conditions of the system dangerously—painful in character. That fatal results may follow such operations, in consequence of reflex impressions made through the fifth nerve upon vital ganglia, is a well-established fact."

The dentist is generally obliged to perform these operations without the benefit to his patient of any of the anæsthetics. The nature of the operations, the necessarily upright position of the patient, the considerable amount of time required to complete them, and the difficulties encountered in the maintenance of anæsthesia, preclude the use of these agents; consequently shock and collapse, in varied degrees, are often the sequel of the severe strain upon the nervous system to which the individual has been subjected. It is not at all uncommon—as I believe every careful observer before me will admit—for strong, robust men, even, to suffer from various symptoms of shock after sitting for two or three hours under the manipulations of the most careful and humane of our profession.

The means at the command of the dentist for the prevention or the mitigation of the suffering incident to dental operations are limited, and known to you all.

The following formulæ have proved very helpful in my practice in relieving the pain attendant upon the preparation of sensitive cavities of decay and preventing nervous exhaustion:

Croton chloral hydrate, grs. x;
Bourbon whiskey, ℥i.
Sig.—Twenty minutes before operating.

Morphia sulph., gr. $\frac{1}{4}$;
Bourbon whiskey, ℥i.
Sig.—Thirty minutes before operating.

Potassium bromide, grs. xx;
Cinnamon water, ℥ii.
Sig.—Thirty minutes before operating.

Potassium bromide, grs. x;
Croton chloral hydrate, grs. x;
Cinnamon water, ℥ii.
Sig.—Thirty minutes before operating.

A wise use of those constitutional remedies, supplemented with the local treatment usually employed, give material aid in relieving the suffering and nervous apprehension of the patient. And if coupled with an educated judgment and quick perception as to the general physical and mental conditions of those presenting themselves for dental operations, and the amount of fatigue and nervous irritation that they are likely to endure with safety, will do much to remove the dangers of such operations, resulting in shock or collapse.

We all have doubtless been more or less guilty of compelling our patients—through carelessness or ignorance of the laws governing the vital forces—to endure the fatigue of long and painful operations when they were by reason of their physical or mental condition, or both, unfit to bear them with impunity.

Children especially should not be compelled to submit to the fatigue and nervous strain of such operations, particularly during the period of rapid growth, puberty, and just preceding it, or while engaged in severe study. At these periods the nervous system is usually taxed to its utmost limit of endurance. When crowded beyond this point the health gives way; and often when too late it is discovered that the foundation has been laid for a train of nervous affections which persist until the end of life.

The practice of filling teeth with gold for children at these periods is, for other reasons than those just now under discussion, not wise, and doubly so when the present and future health of the child is placed in the balance.

The rapid movement of the teeth, by the various methods employed in orthodontia, is often a serious and dangerous operation, on account of the severe and constant irritating influences upon the nerve-centres.

It requires a well-informed mind and the exercise of a clear judgment in order to determine, many times, just what, how, and when this or that operation may be undertaken with safety to the health of the child. It would be much better to sacrifice the personal appearance to the general health ; while, on the other hand, a year or two of schooling might be dispensed with for the sake of the personal appearance. In other words, it would be wisdom to choose health rather than beauty; while a year or two of rest for the child from school-work, to gain the necessary strength to endure the nervous irritation incident to so many cases in regulating the teeth, would be a small sacrifice to make to improve the personal appearance or to place the teeth in a better position to perform the functions for which they were created.

Chlorotic girls and women suffering from uterine and ovarian diseases in their manifold forms, and during gestation, or the functional changes which establish the menopause, are usually in a more or less exalted state of nervous irritability. The nerve-centres seem to be more easily impressed and reflex disturbances are common.

Dental operations of any kind are not well borne by such individuals; but if prolonged and painful in character, they should not be undertaken unless they can be performed through the agency of an anæsthetic or anodynes. In most cases it would be much better to adopt palliative and temporary measures until such time as the health is restored rather than by heroic treatment, to run the risk of nervous exhaustion or more serious consequences. Miscarriage and other serious troubles are not unheard-of *sequelæ* of heroic operations.

Shock from the extraction of teeth is often severe, sometimes inducing alarming symptoms. Syncope is the most common; but this is often followed by a decrease in the surface temperature, cold perspiration, feeble pulse, muscular tremor, nausea, and vomiting. In the more severe cases the depression may be so great as to cause a fatal termination.

Cases of death following the extraction of teeth under the influence of anæsthetics, and sometimes attributed to heart-disease,—fatty degeneration, dilatation, valvular disease, etc.,—are no doubt often the result of shock induced through the progress of the operation before the sensory nerves were completely paralyzed by the action of the anæsthetic. The fifth nerve is considered to be the most sensitive nerve of the body, and it is also among the last to yield to the influences of the anæsthetic drugs. The necessity, therefore, of complete anæsthesia in tooth-extraction, or in any other operation in which this nerve is painfully involved, is self-evident.

The anæsthetic drugs are administered to annihilate pain and prevent shock from the surgical injury; but if they are not carried to the point of profound impression or complete anæsthesia, the desired result is not obtained. The loss of mental consciousness is not anæsthesia, but this condition takes place before the sensory function of the fifth nerve is paralyzed. There is less danger to life in surgical operations from profound than from partial anæsthesia. Partial anæsthesia invites shock, while profound anæsthesia prevents it, as every surgeon and dentist of experience can attest.

Cases are on record, in considerable number, of persons who have died from shock superinduced by mental excitement,—fright, sudden joy, or some great sorrow, the dread of a surgical operation sometimes of even a trivial nature. In many of these cases there was evidence of a weakened condition of the system, most often associated with diseases of the heart.

Dr. George W. Gray, in the "Reference Hand-Book Medical Sciences," vol. vi. p. 439, says, "Lauder Brunton relates a remarkable case of sudden death from shock the result of mental emotion. Some medical students seized the janitor, who had displeased them, and made him believe they intended to behead him. They placed him before a block and blindfolded him, then struck him a sharp blow upon the back of the neck with a wet towel. On removing the bandage they were horrified to find that he was dead."

In the early years of my practice I often operated for individuals—when their time was limited—four, five, and even six hours at a sitting, with only an intermission of a few minutes for luncheon. Looking back upon it now, I cannot refrain from quoting, as applicable to the circumstances, the old familiar line, "Fools rush in where angels fear to tread."

Frequently after such prolonged sittings, particularly when the operations were large gold fillings, the patient has left the chair completely exhausted, and sometimes fainted, presenting marked symptoms of shock, and requiring active measures for resuscitation; while in other cases they have been confined to the bed several days thereafter, from nervous exhaustion.

One case of this character occurred in my practice several years ago, and taught me a valuable lesson.

The history is briefly as follows: J. R., aged fifteen years, health good, but growing rapidly, was brought to me by his mother, three days before he must leave home for boarding school, with the request that his teeth be put in good order. After an examination, it was found that there was so much to be done that a week would be required, with sittings of from two to three hours each day to complete it. The mother insisted upon it being done in the given time, as he could not be late in entering his school. My better judgment was overruled and the work was done, the sitting averaged six hours per day. The boy left the office on the last day very much fatigued. Next day the father called to say that his son was quite ill, and seemed to be laboring under considerable nervous excitement; low nervous fever with great depression followed, which confined him to his bed for a little over four weeks, and he did not enter school until two months after the formal opening.

As further illustrations, I will relate very briefly three other cases from my own practice, one from that of a friend, and three others from the practice of my associate, Dr. Allport.

Mrs. C., aged forty-seven years, in fair health; apparently menopause not yet fully established. Has borne no children. Was operated upon three years ago for the treatment of a chronic alveolar abscess, of ten years standing, by extraction and replantation by the method described in a late number of the *Dental Cosmos*. No anæsthetic was used, as the patient objected to taking it. The operation was well borne, and the lady went home with instructions to return if the tooth gave any annoyance. She did not return. One week after the operation I was sent for, and found her in bed, looking pale and very much exhausted. Her family physician had been in daily attendance since the operation. When called he found her extremities cold, the pulse weak and accelerated, nausea and vomiting, and a very anxious expression of countenance. Restoratives were administered, and after a few hours these symptoms passed away. A low fever followed, from which she recovered slowly. Three months after the operation—which, by the way, was successful, the tooth reuniting with the alveolus without pain or soreness—she left her room for the first time.

The explanation is plain. The nervous system at the time of the operation was taxed to the full extent of endurance by the change taking place in the reproductive functions. The shock of the operation was "the last straw that broke the back" of an already exhausted nervous system. Prostration followed, from which recovery was slow on account of the functional changes which were taking place.

Mrs. L. T. E., a frail-looking, nervous woman, aged twenty-seven years, married five years, and has three living children, the youngest one year old. Health has been delicate since the birth of the last child. Was given a sitting recently for gold filling of one and one-half hours. She was exceedingly nervous and in great dread of the operation.

To obtund sensibility and to quiet the nervous condition the following was administered twenty minutes before operating:

Croton chloral hydrate, grs. x;
Bourbon whiskey, ℥i.
Sig.—Twenty minutes before the operation.

The patient left the office very much fatigued. On arriving home she immediately went to bed, where she was obliged to remain for four days suffering from nervous exhaustion. In this case the operation was not severe nor especially painful.

One-hour sittings for the introduction of plastic fillings have since this time been better borne, but considerable exhaustion followed. The chloral hydrate and whiskey are administered at each sitting, at the earnest request of the patient, and she declares she could not endure the pain without them.

Mr. L. E., a strong, robust-looking man, an operator upon the Chicago Board of Trade, aged fifty-two years, who had just passed through the excitement and anxiety of a corner in wheat in which he had lost heavily, was given a two hours' sitting for a large contour gold operation. On leaving the chair he complained of great depression and faintness. Stimulants were administered, and he soon recovered sufficiently to return home. He afterwards told me that he kept his bed for three days thereafter, being unable to rise on account of the extreme exhaustion.

Several years ago, in a city in central New York, a lady past fifty years of age called upon a professional friend for the extraction of an aching tooth. She was placed in the chair preparatory to the operation, when almost immediately she expired. The post-mortem examination revealed fatty degeneration of the heart.

The coroner's verdict was death from heart-disease, caused by fright. This is an exceptional case; but it illustrates the fact that shock is sometimes superinduced by mental excitement; and that it may under certain weakened conditions of the heart terminate in instant death.

The following cases are from the practice of Dr. Allport:

Mrs. B., aged thirty years, general health good. Pregnant for the fourth time. During each previous pregnancy one or more teeth needed attention, and invariably, when they have been filled with gold requiring extended sittings, nervous prostration has been great; several days being required to recover from it. At the last visit plastic fillings were inserted and the sittings shortened, as the past experience indicated that temporary treatment only was admissible. No prostration followed.

Miss M. G., aged seventeen years, physically frail and exceedingly nervous. Has been under his care since she was nine years old. Teeth very soft. Her record shows sixty-nine fillings have been inserted during this time, all of them a combination of tin and gold. The reasons for this line of treatment were, first, the great saving of fatigue and the nervous energy of the patient,—such fillings can be inserted in one-third of the time required for gold fillings,—and the greater preservative qualities of the combination of metals over gold alone. Had this child been compelled to endure the physical and nervous strain incident to so many gold operations, her health would in all probability have been permanently injured.

Mr. W. D., lawyer, aged fifty years. Strong and robust and of great physical endurance. Had been a regular patient for twenty-five years. Five years ago he was injured by his horse falling upon him, breaking the right femur and fracturing several ribs. The nervous shock was very great, and he never fully recovered from it. Before the accident he had always prided himself upon being able to endure any amount of physical suffering incident to dental operations without flinching. After the accident he was never able to endure a sitting of more than fifteen or twenty minutes long, while during the whole time perspiration would stand in great drops upon his forehead, and he would complain of suffering the most intense agony.

It was only possible to insert temporary fillings from this time until his death, which occurred about two years ago. The fatigue and prostration following even these short sittings was very considerable, and emphasizes the fact that the character of the operations must be tempered to the physical condition of each individual.

Several other marked cases could be recited if time would permit, but these are sufficient for the purposes of illustration.

In the foregoing pages I have endeavored to lay before you the various conditions of surgical shock and collapse, which occasionally result from operations, which to healthy and robust individuals may be of trivial moment, but in those of exalted nervous sensibility, or the physically or mentally overworked, or depressed, result in serious and possibly fatal consequences.

The prevention of such untoward results will depend very largely upon our ability to correctly diagnosticate the physical condition of each individual presenting himself for dental operations; to correctly judge as to the amount of fatigue and suffering each can endure without detriment to health; and the proper use of drugs and other means at our command to mitigate the suffering.

In conclusion let me call your attention to the operator himself.

The dentist of full practice is under a constant nervous and mental strain for from six to eight hours every day. The character of his work requires the closest application, and a skill of no mean degree; the eye must be clear, the hand steady, the brain unclouded. His sensibilities must be under control, his sympathies ready to be drawn upon at any moment to an unlimited extent, his patience must be inexhaustible, his physical endurance and nervous energy that of a Hercules.

I venture to say that the "wear and tear" upon the vital powers of a busy dentist is greater than that of any other class of professional men, the physician and surgeon not excepted. It is granted that the responsibilities which often compass the issues of life and death are much oftener borne by the physician and the surgeon than by the dentist; but, on the other hand, these responsibilities are not being constantly carried; while the difference in the character and surroundings of the cases under treatment gives variety to the work and in a certain sense lightens the load.

In the daily professional life of the dentist there is but little variety. He must perform the same monotonous round of operations, endure the same strain upon already overwrought nerves, the same draught upon his sympathies and patience, and the same tax upon his physical endurance. "'Tis the constant dripping that wears away the stone." Add to these the long hours of close confinement, the stooping positions with contracted chest, the breathing of the poisonous gases of the expired air, and the dangers from disease-infection, and I ask, Is not such a life infinitely more taxing upon the vital energies than that of any other profession?

The fatigue which follows a day's labor, such as I have just indicated, must be experienced to be appreciated. Such labor, continued day after day and week after week, is very exhausting; the appetite fails, digestion and assimilation are impaired, the nerve-centres are disturbed, and unless rest is immediately taken, insomnia or complete nervous prostration is the result.

Some have attempted to recuperate the strength by the use of alcoholic stimulants; but this only places the evil day a few steps further into the future, while, if this course is pursued, the habit grows upon them, and when the final day of reckoning comes the misery is all the greater.

What we need is variety in occupation. I would therefore recommend every dentist to have a "hobby," and ride it well. He should also take frequent periods of recreation with rod or gun, horse or wheel, shell or yacht.

Get out into God's free air; drop all thought of your profession; leave the disagreeables behind you, and become for a little time a boy again!

A half-hour's rest after the mid-day meal is a good thing to indulge in, and if practised every day will prove of very great benefit. Such time is not lost, for with fresh energies more can be accomplished in the same length of time than with powers rendered weak by fatigue.

I fancy some of you are saying, "Don't you find it easier to preach than to practise?" Forsooth, I do! Yet occasionally I practise what I preach by breaking away, as upon this occasion, from my duties and responsibilities, to enjoy the good things of the world; not the least of which is the fellowship of my professional friends.

9 JACKSON STREET.

